# The inhibition of Beclin1-dependent autophagy sensitizes PTC cells to ABT737-induced death

**DOI:** 10.1590/1678-4685-GMB-2022-0170

**Published:** 2024-03-04

**Authors:** Ning Hu, Yanhua Tian, Yanmei Song, Leilei Zang

**Affiliations:** 1 The Second Hospital of Hebei Medical University, Department of General Surgery, Shijiazhuang, Hebei, China.; 2 The Second Hospital of Hebei Medical University, Department of Oncology, Shijiazhuang, Hebei, China.; 3 Hebei People’s Hospital, Shijiazhuang, Department of Infection Management/Public Health, Hebei, China.

**Keywords:** ABT737, Beclin1, autophagy, apoptosis, papillary thyroid carcinoma

## Abstract

ABT737 is used as a specific BCL2 inhibitor, which can treat papillary thyroid carcinoma (PTC). However, the effect of ABT737 on PTC cell apoptosis is limited. Moreover, BCL2 inhibition causes the activation of Beclin1-dependent autophagy. Our study aimed to explore the effects of autophagy and Beclin1 on ABT737 efficacy in PTC. The experimental data showed that ABT737 synchronously enhanced autophagic activity and apoptosis level in PTC cells. ABT737 also promoted the dissociation of BCL2-Beclin1 and BCL2-Bax complexes. Autophagy inhibitors, Bafilomycin A1 and 3-MA, enhanced the inhibitory effect of ABT737 on the survival and function in PTC cells. Consistently, autophagy inhibition with Beclin1 pharmacological inhibitor (spautin-1) also enhanced the efficacy of ABT737. Additionally, ABT737 at low-dose promoted LC3 conversion in PTC cells, and did not affect PTC cell apoptosis and survival. However, The efficacy of low-dose of ABT737 in PTC cell apoptosis and survival was displayed with the addition of Bafilomycin A1, 3-MA or spautin-1. In conclusion, the limited role of ABT737 in PTC cell apoptosis is attributed to its promoting effect on Beclin1-dependent autophagy. Therefore, autophagy inhibition based on Beclin1 downregulation can enhance the sensitivity of PTC cells to ABT737-induced death.

## Introduction

The incidence rate of papillary thyroid carcinoma (PTC) is the highest in thyroid cancer patients, reaching 70-80% (Ertek et al., 2012). The standard treatment for PTC includes surgery, radioiodine therapy, and thyrotropin suppression. Most patients responded well to the above treatments (Vaccarella et al., 2015). However, patients with recurrent PTC, more aggressive subtypes, or anaplastic thyroid cancer (ATC) have a poor course of disease. Finding improved treatment strategies is necessary to deal with these thyroid cancers with poor prognosis (Nagaiah et al., 2011; Hsu et al., 2014).

ABT737 is a synthetic BH3 mimic molecule with high affinity in binding to BCL2, BCL-XL and BCL-W, thereby dissociating the pro-apoptotic molecules, Bax and Bak, and leading to the initiation of apoptosis (Park et al., 2013; Packer et al., 2019; Tailler et al., 2019). ABT737 can promote the apoptosis of various malignant tumors (Park *et al.*, 2013; Packer *et al.*, 2019; Tailler *et al.*, 2019). Accordingly, ABT737 is regarded as a promising anti-cancer treatment. ABT737 can also promote the apoptosis in PTC cells (Gunda et al., 2017). However, its single application lacks sufficient sensitivity in the treatment of PTC. Previous study has shown that 2 μM of ABT737 alone can only increase the apoptosis level in PTC cell line, BCPAP, to a small extent, while not affecting the apoptosis of another PTC cell, TPC-1 (Gunda *et al.*, 2017). However, the addition of related intervention enhances the pro-apoptotic ability of ABT737 (Gunda *et al.*, 2017). Therefore, finding a combination scheme to cooperate with the pro-apoptotic ability of ABT737 in PTC is an effective channel to improve ABT737 efficacy.

BCL2 inhibition can also dissociate the BCL2-Beclin1 complex, thereby releasing the autophagy-stimulating factor Beclin1, which causes its entry into the autophagy flux and activates autophagy (Sinha and Levine, 2008; Kang et al., 2011). A previous study demonstrated that the intervention of ABT737 can lead to the reduction in the interaction between Beclin1 and BCL2, and promote the autophagic response (Pedro et al., 2015). As a protective mechanism, autophagy has an anti-apoptotic effect. Autophagy is proven to prevent cancer cells from apoptosis (Fitzwalter and Thorburn, 2015). Autophagy downregulation enhances apoptotic sensitization of cancer cells by inhibiting the transformation of FOXO3a (Fitzwalter *et al.*, 2018). Moreover, autophagy suppresses TNFα-induced apoptosis, which prevents tumors from T cell-mediated cytotoxicity (Young et al., 2020). Therefore, we speculate that Beclin1-dependent autophagy promoted by ABT737 may offset ABT737-induced apoptosis in PTC cells. If the above hypothesis is established, the inhibition of Beclin1-dependent autophagy may sensitize ABT737-caused PTC cell apoptosis, and become an effective adjuvant strategy for ABT737 treatment.

In this study, autophagic and apoptotic parameters were detected upon ABT737 intervention, and ABT737 and autophagic or Beclin1 pharmacological inhibitors were jointly applied. Our experimental data revealed for the first time the effective auxiliary mean of ABT737 in PTC treatment.

## Material and Methods

### Cell lines and cell culture

Human PTC cell lines (BCPAP and TPC-1) were purchased from the cell bank of Chinese Academy of Sciences. The above cells were cultured in RPMI-1640 medium (Invitrogen) and 10% fetal bovine serum (FBS, Invitrogen). All cells were stored in a humid environment at 37 °C and 5% CO_2._

### Western blotting assays

The whole lysates from cells or tissues were extracted using ice-cold RIPA lysis buffer. The protein content was measured using BCA protein quantitative kit in accordance with manufacturer’s protocols (Thermo Fisher Scientific, MA, USA). After being blocked with 5% non-fat milk for 1 hour at room temperature, PVDF membranes were incubated with the antibodies against LC3 (#3868, 1:1000), Cleaved-caspase3 (#9664, 1:1000), BCL2 (#15071, 1:1000), Beclin1 (#4122, 1:1000), Bax (#41162, 1:1000), PARP (#9532, 1:1000) and GAPDH (Cell Signaling Technology, MA, USA) overnight at 4 ˚C. Subsequently, the HRP-conjugated secondary antibody (#SA00001-1 and #SA00001-2, 1:5000; Proteintech, Wuhan, China) was applied for 1 hour at room temperature. The bands were visualized using enhanced chemiluminescent substrate reagent kit (Amersham; Cytiva) and chemiluminescence system (Amersham Image 600; General Electric; Cytiva). The signal densitometry was semi-quantified using ImageJ software (v1.8.0).

### Co-immunoprecipitation (Co-IP) assays

Total proteins were extracted using RIPA lysis and extraction buffer. Next, we used 100 μL of ice buffer to rinse the beads, added 100 μL of antibody-binding buffer, rotated the antibody and magnetic beads for 30 minutes, and then used 200 μL of buffer to rinse the beads. Lysates and antibody-binding magnetic beads were incubated at room temperature for 1 hour and washed with 200 μL of buffer. The beads were washed with 20 μL of elution buffer to remove the supernatant. Lysates were extracted for Co-IP using anti-BCL2 antibody (#15071, 1:50, Cell Signaling Technology), and then Western Blotting assays using anti-Beclin1, Bax and BCL2 antibodies were applied to observe the precipitates.

### Cellular immunofluorescence assays

Cells cultured on 6-cm dishes received the corresponding treatment. The treated cells were fixed using 4% paraformaldehyde (PFA). After fixation, cells were blocked using 1% BSA, and incubated with the indicated primary antibodies (LC3, #3868, 1:2000, Cell Signaling Technology) at 4 °C overnight. Then, the indicated cells were stained with ﬂuorochrome-labelled secondary antibody for 1 h and then counterstained with DAPI for 10 min. Ultimately, the cells were observed and recorded under the Leica confocal microscope (Leica Microsystems, Frankfurt, Hessen, GER).

### Analyses of cell apoptosis

Cell apoptosis was assessed using (1) Annexin V-FITC/PI (AV/PI) staining: the indicated cells were collected, and then cell staining was performed according to manufacturer’s protocols (Thermo Fisher Scientific). Then, apoptotic cells were evaluated using flow cytometer (Cytomics FC500, Beckman Coulter, Florida, USA). (2) Caspase3 activity: Caspase3 activity was detected by ApoAlert caspase fluorescent assay kit (Clontech, CA, USA). The treated cells placed on 6-well plates were lysed in 120 μL of lysis buffer, and incubated on ice for 10 minutes. 120 μL of reaction buffer containing 12 μL of caspase3 fluorescent substrate (1 mM) were added to each well and incubated for 1 hour at 37 °C. The fluorescent intensity was quantified using the fluorospectrophotometer (Synergy2, BioTek, VT, USA; excitation at 400 nm and emission at 505 nm). The cells intervened by Caspase3 inhibitor (DEVD-CHO, MedChemExpress, NJ, USA) were considered a negative control to exclude the nonspecific hydrolysis of the substrate.

### Analyses of total cell death

To assess the total death level, trypan blue exclusion assays were carried out as previously described (Ni et al., 2014). The cells failing to exclude the presented blue-dye were defined as the dead cells. The total death rate (%) = number of dead cells / (number of living cells + number of dead cells) × 100%.

### Analyses of cell proliferation

To evaluate cell proliferation, cell counting Kit-8 (CCK-8) assays were performed using the related kit (Dojindo, Kumamoto, Japan) in accordance with manufacturer’s protocols. For CCK-8 assay, the corresponding cells were plated into 96-well plates with 2,500 cells/well. After the indicated time, all cells were incubated with 10 μL of CCK-8 reagent. After 2 hours of incubation, the optical density at 450 nm (OD450) was measured using Varioskan Flash reader (Thermo Fisher Scientific).

### Analyses of cell migration

Cell migration was measured by transwell assays: corresponding reagents-treated cells suspended in serum-free DMEM were seeded onto the upper chamber of Transwell inserts (24-well inserts, Millipore, MA, USA). DMEM containing 20% FBS was added to lower chamber. After 36 hours of incubation, the cells migrating into lower surface of the inserts were fixed, stained with 1% crystal violet, and photographed (Olympus Corporation). The migratory level was evaluated by counting the number of stained cells. 

### Statistical analysis

GraphPad Prism software 8 was used for statistical analysis. One-way ANOVA or two-way ANOVA test were applied for comparison. Bonferroni test was used for post-hoc multiple comparisons of ANOVA test. P value < 0.05 was considered statistically significant.

## Results

### Treatment of ABT737 upregulated apoptotic and autophagic parameters in PTC cells

**Figure 1.**
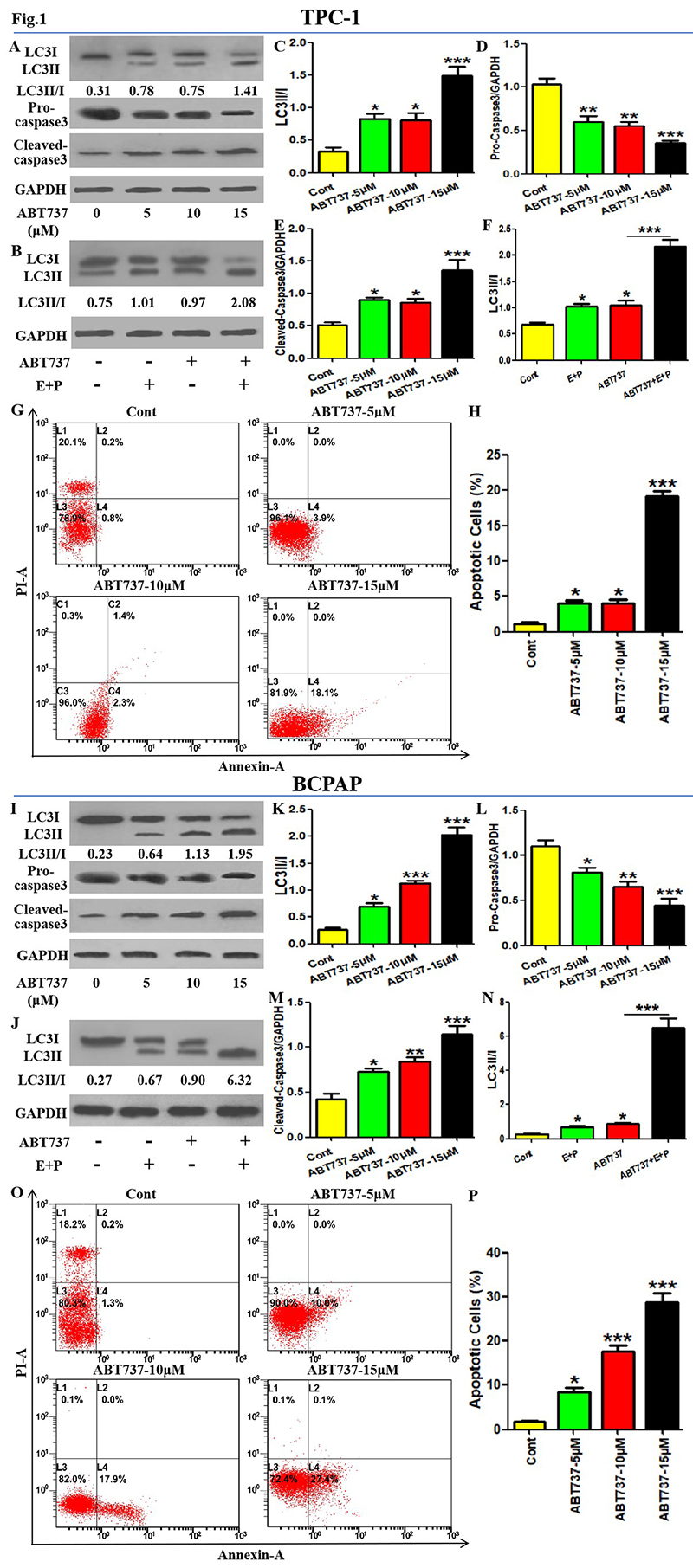
Treatment of ABT737 upregulated apoptotic and autophagic parameters in PTC cells. (A, C-E) After treatment with different concentrations of ABT737 (0,5,10, 15 μM) for 8 hours, the protein expression of LC3, pro-caspase3 and Cleaved-caspase3 in TPC-1 cells were detected using western Blotting assays. LC3 conversion rate is defined as LC3II/I ratio. (B, F) After treatment with ABT737 (10 μM) or/and E64D plus Pepstain A for 8 hours, LC3 protein expression in TPC-1 cells were detected using Western Blotting assays. LC3 conversion rate is defined as LC3II/I ratio. (G-H) After treatment with different concentrations of ABT737 for 24 hours, cell apoptosis level was evaluated using AV/PI staining (Annexin-A-positive cells are considered apoptotic cells). LC3 conversion rate is defined as LC3II/I ratio. (I-P) The treatment and detection as described in (A-H) were repeated in BCPAP cells. The experiments were repeated on three samples. Data are presented as mean±SEM from three independent assays. *P<0.05; ***P<0.001. Cont, control group; E, E64D; P, Pepstain A.

The effects of ABT737 on autophagy and apoptosis in PTC cells need to be investigated. As shown in Figure 1 A , C-E, all concentrations of ABT737 increased LC3 conversion rate (LC3II/I ratio) and cleaved-caspase3 expression level in TPC-1 cells, and decreased pro-caspase3 expression level, among which 15 μM of ABT737 has the most obvious effects. Moreover, ABT737 increased the number of apoptotic cells at different concentrations, which was the most effective at the highest concentration (Figure 1 G  -H). Furthermore, ABT737 promoted LC3 conversion in TPC-1 cells in the presence or absence of lysosomal protease inhibitors (E64d + Pepstatin A) (Figure 1B, F). Similar to those of TPC-1 cells, ABT737 upregulated LC3 conversion, cleaved-caspase3 expression and apoptotic cells, and downregulated pro-caspase3 expression in BCPAP cells in a concentration-dependent manner (Figure 1I-P). In addition, the addition of lysosomal protease inhibitors upregulated LC3 conversion in two PTC cells, which indicated the stability of autophagy flux and the effectiveness of our experimental system (Figure 1 B ,E,J,N). These results suggest that ABT737 promotes both autophagic activity and apoptosis level in PTC cells.

### Treatment of ABT737 inhibited the co-immunoprecipitation level of BCL2 and Beclin1/Bax in PTC cells

**Figure 2.**
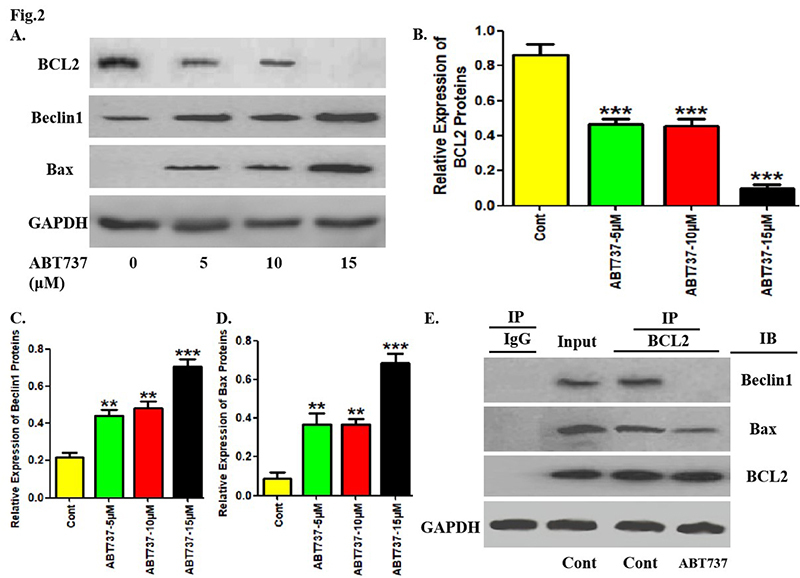
Treatment of ABT737 inhibited the co-immunoprecipitation level of BCL2 and Beclin1/Bax in PTC cells. (A) After treatment with different concentration of ABT737 for 8 hours, the protein levels of BCL2, Beclin1 and Bax in TPC-1 cells were detected using western blotting assays. (B-D) The histogram represents the relative expression of each protein in **A** (the ratio of each protein to GAPDH). (E) TPC-1 cells were treated with ABT737 for 8 hours. The lysates of corresponding cells were extracted with anti-BCL2 antibody for co-immunoprecipitation, and then the precipitation was detected by Western Blotting assays with anti-Beclin1, Bax and BCL2 antibodies. The experiments were repeated on three samples. Data are presented as mean±SEM from three independent assays. **P<0.01; ***P<0.001. Cont, control group; IP, the antibody for immunoprecipitation; IB, the antibody for immunoblot.

Then, the effects of ABT737 on pro-autophagic molecule and pro-apoptotic molecule in PTC cells were observed. As shown in Figure 2 A,B, the application of ABT737 gradually reduced BCL2 protein expression in TPC-1 cells. Moreover, different concentrations of ABT737 increased the protein expression of Beclin1 and Bax in TPC-1 cells, which was the most significant at the highest concentration (Figure 2 A ,C,D). Of note, ABT737 administration significantly inhibited the co-immunoprecipitation levels of BCL2 with Beclin1 and BCL2 with Bax in TPC-1 cells (Figure 2 E ).

### Autophagy inhibitors enhanced the inhibition of ABT737 on the survival and migration in PTC cells

**Figure 3.**
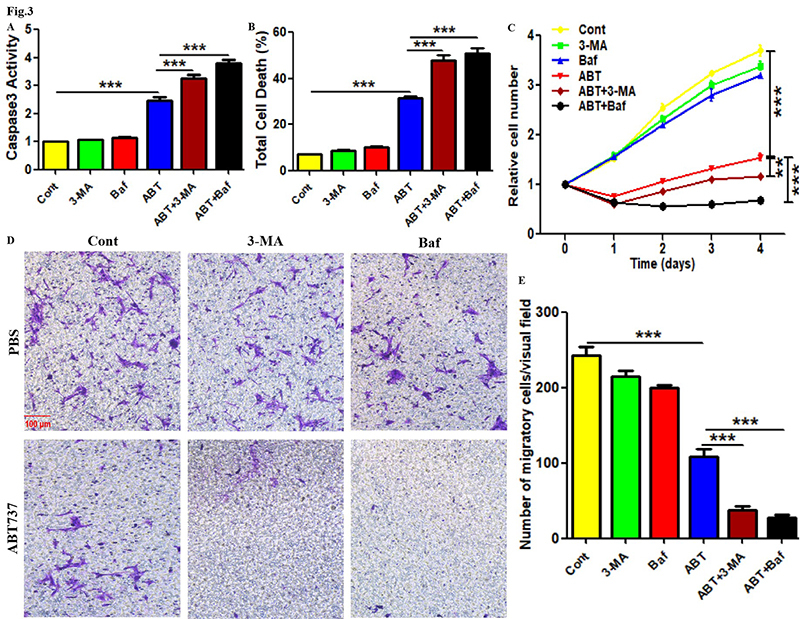
Autophagic inhibitors enhanced the inhibition of ABT737 on the survival and migration in PTC cells. (A) After treatment with ABT737 along with or without 3-MA or Bafilomycin A1 for 1 day, the apoptosis level in BCPAP cells was assessed by detecting Caspase3 activity with corresponding experimental kit. (B) After treatment with ABT737 along with or without 3-MA or Bafilomycin A1 for 1 day, the total death level in BCPAP cells was measured using trypan blue staining. (C) After treatment with ABT737 along with or without 3-MA or Bafilomycin A1 for given time, the proliferative level in BCPAP cells was measured using CCK-8 assays. (D,E) After treatment with ABT737 along with or without 3-MA or Bafilomycin A1 for 36 hours, the migratory level in BCPAP cells was measured using Transwell assays. Scale bar: 100 μm. The experiments were repeated on three samples. Data are presented as mean±SEM from three independent assays. **P<0.01; ***P<0.001. Cont, control group; Baf, Bafilomycin A1; ABT, ABT737.

The role of autophagy in ABT737-regulated PTC cell death also needs to be clarified. As shown in Figure 3 A  -B, although the addition of 3-MA or Bafilomycin A1 did not directly affect Caspase3 activity and total death level, they enhanced the upregulation of ABT737 on Caspase3 activity and total death level in BCPAP cells. In addition, the application of 3-MA or Bafilomycin A1 had no significant effect on the proliferation and migration in BCPAP cells, and ABT737 administration significantly inhibited the above parameters (Figure 3 C  -E). However, the addition of 3-MA or Bafilomycin A1 augmented the inhibitory effect of ABT737 on the proliferation and migration in BCPAP cells (Figure 3 C  -E). Considering that the detection of cell migration in this study was based on cell viability, cell migratory level still reflects cell proliferative activity. This suggests the significance of autophagy in PTC cell apoptosis regulated by ABT737.

### Beclin1 inhibitor promoted the inhibition of ABT737 on the survival and migration in PTC cells

**Figure 4.**
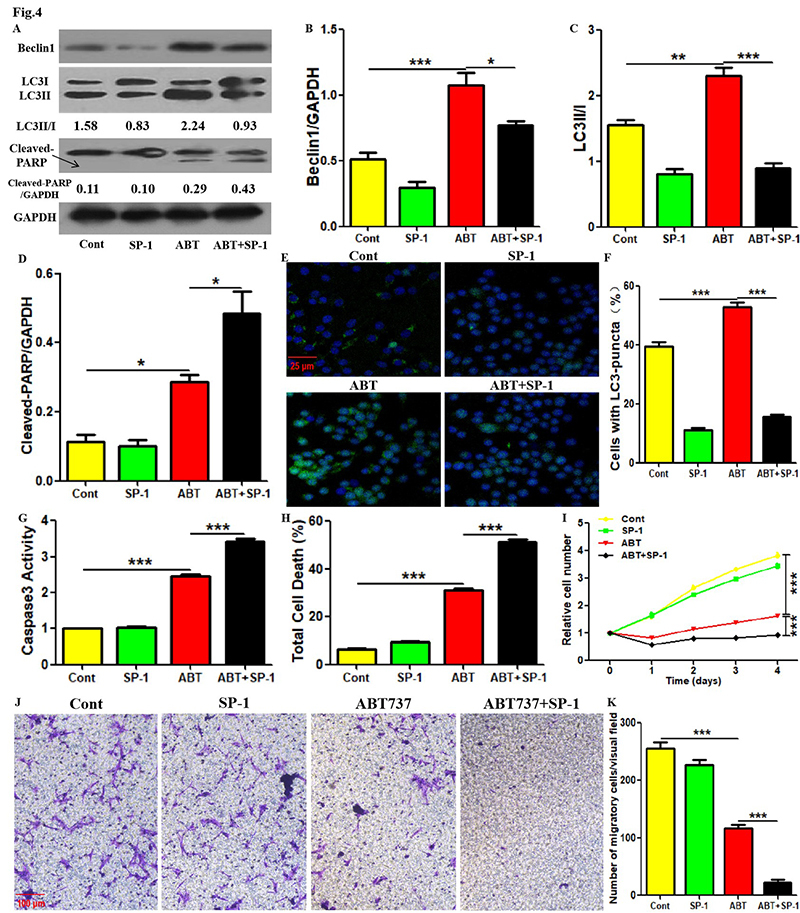
Beclin1 inhibitor promoted the inhibition of ABT737 on the survival and migration in PTC cells. (A-D) After treatment with ABT737 along with or without spautin-1 for 8 hours, the protein levels of Beclin1, LC3 and Cleaved-caspase3 in TPC-1 cells were detected using western blotting assays. LC3 conversion rate is defined as LC3II/I ratio. (E, F) After treatment with ABT737 along with or without spautin-1 for 12 hours, the LC3-puncta were imaged via immunofluorescence staining and observed under confocal microscope. Scale bar, 25 μm. The cells containing more than 5 LC3-puncta were defined as positive cells. (G) After treatment with ABT737 along with or without spautin-1 for 1 day, the apoptosis level in TPC-1 cells was assessed by detecting Caspase3 activity with corresponding experimental kit. (H) After treatment with ABT737 along with or without spautin-1 for 1 day, the total death level in TPC-1 cells was measured using trypan blue staining. (I) After treatment with ABT737 along with or without spautin-1 for given time, the proliferative level in TPC-1 cells was measured using CCK-8 assays. (J, K) After treatment with ABT737 along with or without spautin-1 for 36 hours, the migratory level in TPC-1 cells was measured using transwell assays. Scale bar: 100 μm. The experiments were repeated on three samples. Data are presented as mean±SEM from three independent assays. ***P<0.001. Cont, control group; SP-1, spautin-1; ABT, ABT737.

Next, the role of Beclin1 in PTC cell death regulated by ABT737 also needs to be identified. As shown in Figure 4 A  -C, E-F, Beclin1 inhibitor spautin-1 (Liu et al., 2011) not only inhibited Beclin1 expression, LC3 conversion and LC3-puncta formation, but also blocked ABT737-upregulated Beclin1 level, LC3 conversion and LC3-puncta formation in TPC-1 cells. In addition, although spautin-1 administration did not affect cleaved-PARP expression, Caspase3 activity and total death level, it promoted the upregulation of ABT737 on Cleaved-PARP expression, Caspase3 activity and total death level in TPC-1 cells (Figure 4 A ,D,G,H). Moreover, ABT737 application inhibited the proliferation and migration in TPC-1 cells, which was enhanced with the addition of spautin-1 (Figure 4 I  -K). The above results show the significance of Beclin1 in PTC cell autophagy and death regulated by ABT737.

### Low-dose of ABT737 also activated PTC cell autophagy

**Figure 5.**
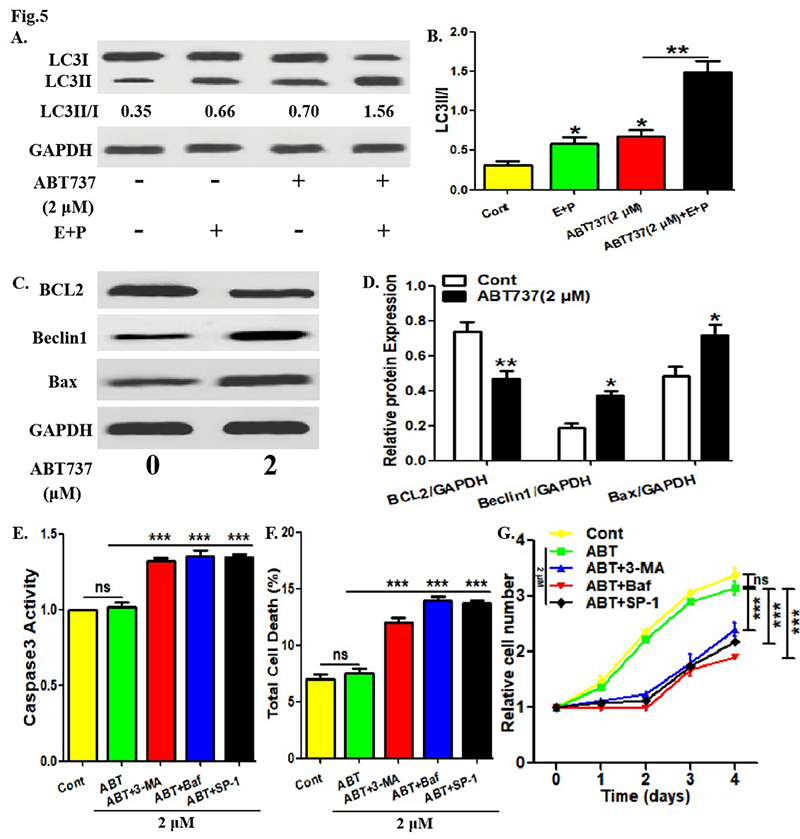
Low-dose of ABT737 also activated PTC cell autophagy. (A, B) After treatment with ABT737 (2 μM) or/and E64D plus Pepstain A for 8 hours, LC3 protein expression in TPC-1 cells were detected using Western Blotting assays. LC3 conversion rate is defined as LC3II/I ratio. (C, D) After treatment with ABT737 (2 μM) for 8 hours, the protein levels of BCL2, Beclin1 and Bax in TPC-1 cells were detected using Western Blotting assays. (B-D) The histogram represents the relative expression of each protein in **C** (the ratio of each protein to GAPDH). (E) After treatment with ABT737 (2 μM) along with or without 3-MA, Bafilomycin A1 or spautin-1 for 1 day, the apoptosis level in TPC-1 cells was assessed by detecting Caspase3 activity with corresponding experimental kit. (F) After treatment with ABT737 along with or without 3-MA, Bafilomycin A1 or spautin-1 for 1 day, the total death level in TPC-1 cells was measured using trypan blue staining. (G) After treatment with ABT737 along with or without 3-MA, Bafilomycin A1 or spautin-1 for given time, the proliferative level in TPC-1 cells was measured using CCK-8 assays. The experiments were repeated on three samples. Data are presented as mean±SEM from three independent assays. *P<0.05; **P<0.01; ***P<0.001. Cont, control group; E, E64D; P, Pepstain A; ABT, ABT737; Baf, Bafilomycin A1; SP-1, spautin-1.

Although the promoting effect of ABT737 on the protective autophagy in PTC cells was demonstrated, a low-dose of ABT737 (2 μM) was used in previous study. Therefore, we also need to confirm the regulatory effects of 2 μM of ABT737 on the corresponding parameters in TPC-1 cells. As shown in Figure 5 A ,B, ABT737 promoted LC3 conversion in TPC-1 cells in the presence or absence of lysosomal protease inhibitors, and the addition of lysosomal protease inhibitors enhanced LC3 conversion in TPC-1 cells. Furthermore, 2 μM of ABT737 decreased BCL2 protein expression and increased Beclin1 and Bax protein expression in TPC-1 cells (Figure 5 C  -D). The above data indicate that ABT737 at low-dose can activate autophagy and promote autophagic and apoptotic protein levels. Subsequently, it was observed that 2 μM of ABT737 did not affect Caspase3 activity in TPC-1 cells, but the addition of 3-MA, Bafilomycin A1 or spautin-1 enhanced the efficacy of 2 μM of ABT737 (Figure 5 E ). Consistent with this, 2 μM of ABT737 did not affect total death level and proliferation in TPC-1 cells, but the addition of 3-MA, Bafilomycin A1 or spautin-1 enhanced the efficacies of 2 μM of ABT737 (Figure 5 F ,G).

## Discussion

ABT737 can promote the apoptosis of cancer cells by competitively inhibiting the anti-apoptotic molecule BCL2, which makes ABT737 available in the treatment of PTC (Gunda et al., 2017). Nevertheless, ABT737 did not sensitize PTC cells to apoptosis (Gunda *et al.*, 2017). The competitive inhibition of BCL2 by ABT737 can also promote Beclin1-dependent autophagy (Pedro et al., 2015), which leaves an interesting scientific question: whether Beclin1-dependent autophagy is the factor to desensitize ABT737 to PTC cell apoptosis. First of all, our experimental data clarified that ABT737 can lead to enhanced apoptotic level and autophagic response in PTC cells. Remarkably, previous study showed that ABT737 administration is ineffective in altering the apoptosis in TPC-1 cells (Gunda *et al.*, 2017). Here, we increased the intervention concentration of ABT737, which leads to the discrepancy between our experimental data and previous study (Gunda *et al.*, 2017). Additionally, due to the inhibition on BCL2 level, ABT737 administration also caused the dissociation of BCL2-Beclin1 and BCL2-Bax complexes in PTC cells, which resulted in the simultaneous increase of Beclin1 and Bax protein levels. Accordingly, it can be inferred that ABT737 leads to the release of Beclin1 and Bax by binding to BCL2, thereby enhancing the autophagy and apoptosis in PTC cells, respectively.

Through the application of autophagy inhibitors, it was found that autophagy inhibition enhanced ABT737-promoted death and -inhibited survival in PTC cells. Previous studies have shown that autophagy, as an intracellular homeostasis-maintaining mechanism, can resist apoptosis, especially in cancer cells (Fitzwalter and Thorburn, 2015; Fitzwalter *et al.*, 2018; Young et al., 2020). Based on the promotion of ABT737 on autophagy and Beclin1’s dissociation, the insensitivity of ABT737 in PTC cell apoptosis may be due to the effect of Beclin1-dependent autophagy. Our data confirmed that autophagy inhibition and ABT737 have additive effect on promoting PTC cell apoptosis, which indicates the regulatory effect of autophagy on apoptosis alteration under ABT737 intervention. Notably, the two autophagy inhibitors used above, 3-MA and Bafilomycin A1, inhibited the formation of autophagosomes and the fusion of autolysosomes, respectively, and are not specifically targeting Beclin1. As a pharmacological inhibitor of Beclin1, spautin-1 is more suitable for studying the relationship between autophagy and ABT737 efficacy. As expected, spautin-1 administration not only reversed ABT737-promoted PTC cell autophagy, but also amplified ABT737-promoted apoptosis and -inhibited survival in PTC cells. The above results demonstrated the significance of Beclin1-dependent autophagy in PTC cell apoptosis regulated by ABT737, and suggested that the inhibition of Beclin1-dependent autophagy may be an effective combination scheme to enhance ABT737 efficacy in PTC. Due to the low-dose level of ABT737 used in previous PTC-related study (2 μM) (Gunda et al., 2017), we ensured that the promoting effect of ABT737 on PTC cell autophagy was also replicated at low-dose level. As expected, ABT737 at low-dose promoted LC3 conversion in PTC cells. Nevertheless, ABT737 at low-dose had no effect on PTC cell apoptosis and survival. From the current findings that ABT737 at low-dose simultaneously enhanced autophagic and apoptotic protein levels in PTC cells, it was further inferred that the protective autophagy caused by ABT737 offsets its pro-apoptotic function. The reinforcement of low-dose of ABT737 efficacies by corresponding reagents with autophagy-activating function confirms our inference.

**Figure 6.**
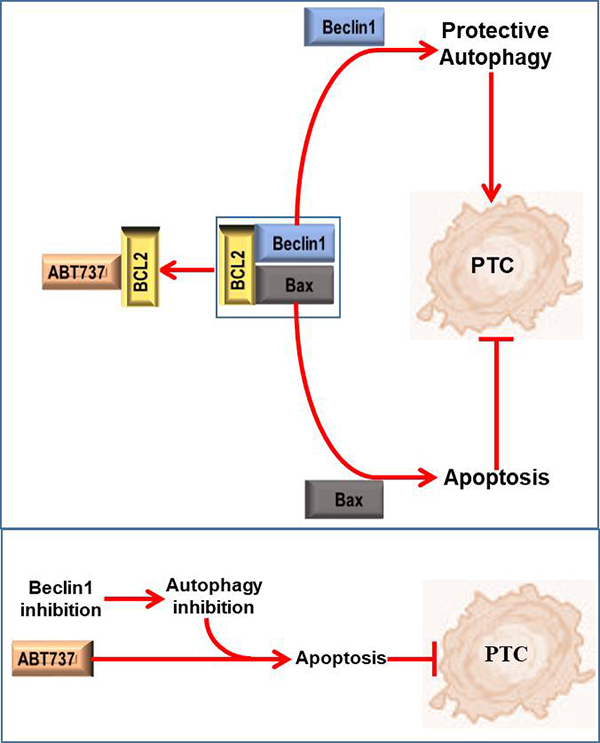
Relationship pattern between Beclin1-dependent autophagy and ABT737 in PTC cells under ABT737 intervention. ABT737 can competitively bind to BCL2, thereby dissociating Beclin1 and Bax from BCL2-Beclin1 complex and BCL2-Bax complex in PTC cells, respectively. The release of Bax can promote apoptosis and repress PTC carcinogenesis. However, the release of Beclin1 can promote protective autophagy, which is a resistance factor of ABT737 efficacy. Accordingly, the inhibited autophagy caused by Beclin1 inhibition can cooperate with the role of ABT737 in promoting PTC cell apoptosis and inhibiting PTC carcinogenesis.

In conclusion, our data suggest that ABT737 can release Beclin1 and Bax by binding to BCL2, thus promoting the protective autophagy and apoptosis in PTC cells, which causes the limited role of ABT737 in PTC cell apoptosis. Furthermore, under ABT737 intervention, the activation of Beclin1-dependent autophagy is not only an adverse factor of ABT737 efficacy in PTC, but also a target for the joint strategy to enhance ABT737 efficacy. Our working model is described in Figure 6. Our research provides more theoretical basis for optimizing the treatment strategy of PTC.
