# Erratum: The inhibition of Beclin1-dependent autophagy sensitizes PTC cells to ABT737-induced death

**DOI:** 10.1590/1678-4685-GMB-2022-0170er

**Published:** 2026-04-20

In the article “The inhibition of Beclin1-dependent autophagy sensitizes PTC cells to ABT737-induced death”, with DOI code number: https://doi.org/10.1590/1678-4685-GMB-2022-0170, published in the journal Genetics and Molecular Biology, 47(1):e20220170, 2024, in the page 4:

**Where it was written:**

**Figure:**
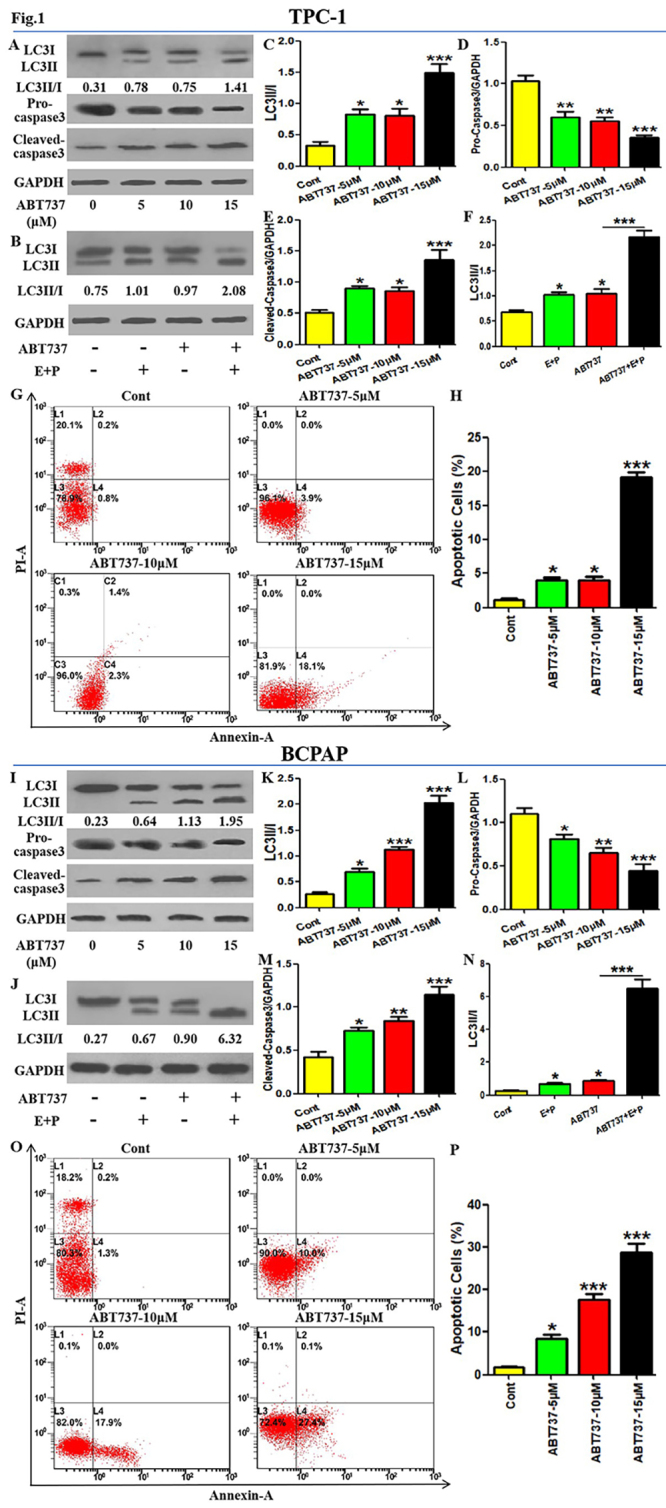

**Should read:**

**Figure:**
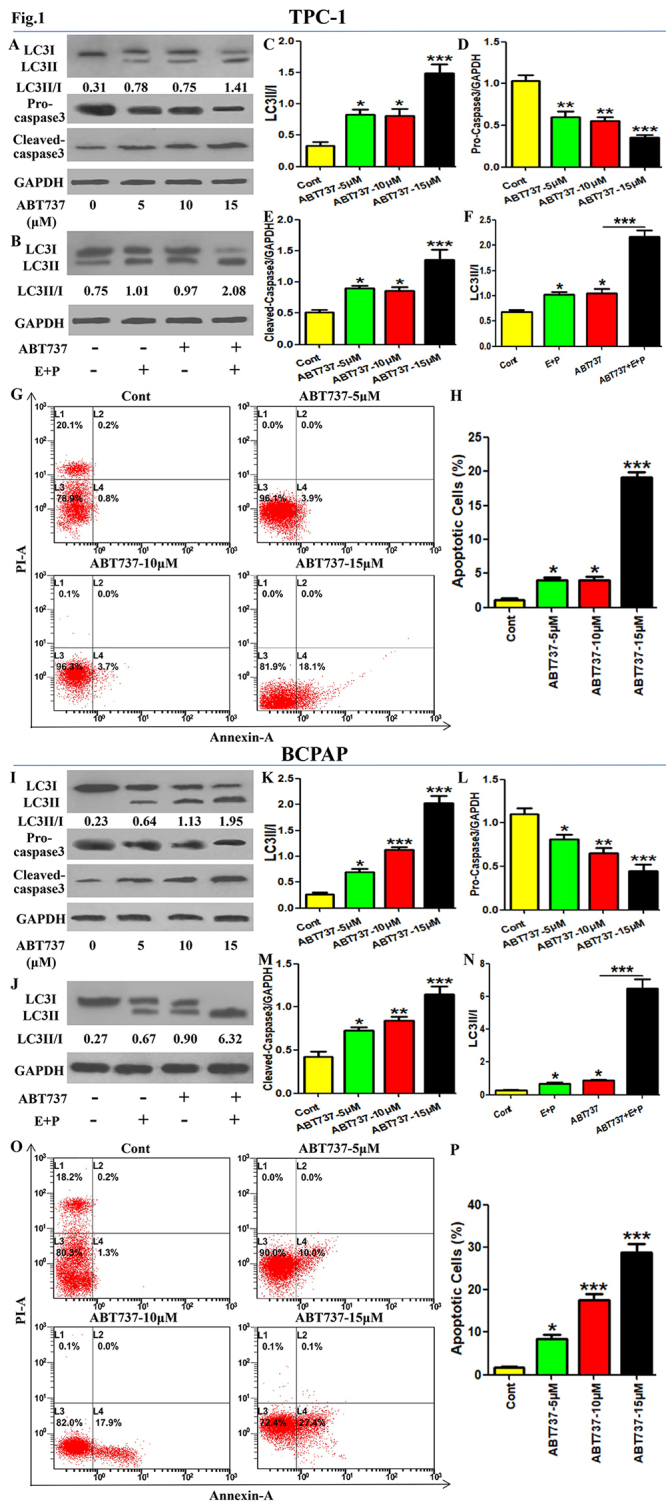

Prof. Dr. Augusto Schrank

Prof. Dr. Carlos F. M. Menck

Prof. Dr. Marcia Pinheiro Margis

Editor-in-chief

